# Superposed picosecond luminescence kinetics in lithium niobate revealed by means of broadband fs-fluorescence upconversion spectroscopy

**DOI:** 10.1038/s41598-020-68376-6

**Published:** 2020-07-09

**Authors:** A. Krampf, S. Messerschmidt, M. Imlau

**Affiliations:** School of Physics, Osnabrueck University, Barbarastrasse 7, 49076 Osnabrueck, Germany

**Keywords:** Optics and photonics, Nonlinear optics, Ultrafast photonics

## Abstract

Various manifestations of small polarons strongly affect the linear and nonlinear optical properties of the oxide crystal lithium niobate ($\hbox {LiNbO}_3$, LN). While related transient absorption phenomena in LN have been extensively studied in recent decades, a sound microscopic picture describing the blue-green (photo)luminescence of lithium niobate single crystals is still missing. In particular, almost nothing is known about: (i) the luminescence build-up and (ii) its room temperature decay. We present here the results of our systematic experimental study using nominally undoped and Mg-doped LN crystals with different Mg concentration. Picosecond luminescence was detected by means of femtosecond fluorescence upconversion spectroscopy (FLUPS) extended to the inspection of oxide crystals in reflection geometry. Two distinct luminescence decay components on the picosecond time scale are revealed. While a short exponential decay is present in each sample, a longer non-exponential decay clearly depends on the crystal composition. Since transient absorption spectroscopy excludes geminate small polaron annihilation as microscopic cause of the luminescence, both decay components are discussed in the context of self-trapped exciton (STE) transport and decay.

## Introduction

$\hbox {ABO}_3$ perovskite-like ferroelectrics exhibiting unique electronic properties^1^ are important materials for optical frequency converters^2^, THz pulse generation^3^ or promise advances in the fields of photovoltaics^4^, oxide electronics^5^ and electroluminescent devices^6^. In these materials, strong charge carrier-phonon coupling often lead to pronounced photo-induced polaronic effects manifesting in, e.g., laser-induced bulk damage^7,8^, and broad photoluminescence bands emitted from $\hbox {BO}_6$ octahedra^9–11^.

Lithium niobate is a frequently studied example of ferroelectric perovskite-like oxides, known for its rich defect structure leading to a conglomeration of intrinsic and extrinsic small polarons^7^. Whereas light-induced phenomena in LN, such as transient absorption, have been extensively studied in recent decades relating them with formation and transport of small polarons^12–23^, a sound microscopic picture describing the blue-green (photo)luminescence of lithium niobate single crystals is still missing.

While recently geminate small polaron annihilation has been proposed to lead to a two component luminescence decay in Mg-doped lithium niobate at low temperatures^24^, Messerschmidt *et al.* showed that low temperature luminescence and absorption decay on very different time scales^25^. They revived the idea of radiatively decaying self-trapped excitons located at niobium-oxygen octahedra, which has been introduced over thirty years ago in the pioneering work of Blasse *et al.*^9,26–29^. They concluded that an effect of STE formation on the light-matter interaction at elevated temperatures could per se no longer be neglected. In fact, no absorption feature of intrinsic self-trapped excitons has been reported so far, while Messerschmidt *et al.* related long-lived STEs pinned on extrinsic defects with a transient absorption in the blue-green spectral range^25^. These transients, which can last from seconds to hours, should naturally have a major influence on light-induced damage of the crystals, especially when illuminated with continuous wave lasers or short laser pulses at high repetition rates.

In their revised excitation and relaxation model, different excitation paths entailing pinned-STE formation are proposed, which have recently been confirmed experimentally for Fe-doped crystals^30^. One of these paths is the band-to-band generation of electron-hole-pairs subsequently forming self-trapped excitons, which in turn migrate through the crystal until they are pinned on a defect. Therefore, the STE formation and decay times are important characteristics regarding the extend to which the propagation of ultrashort laser pulses is affected by the (pinned-)STE absorption features, especially at elevated temperatures where STE migration and pinning could play a dominant role. The authors showed that the temperature-dependent luminescence peak position deviates from the Varshni behavior for temperatures above $\approx 200\,\hbox {K}$^25^. Room temperature luminescence kinetics might therefore not be assessable from low temperature data via extrapolation.

For a comprehensive understanding of fs-laser pulse propagation in the presence of STEs and moreover of photo-excited charge carrier kinetics in lithium niobate in general, two important aspects have not yet been (sufficiently) investigated due to lack of temporal resolution^24–26,31–34^: (i) luminescence build-up and (ii) room temperature decay. The second point is particularly important with regard to applications. The self-trapped exciton formation time, on the other hand, helps to clarify whether small polarons and self-trapped excitons in lithium niobate are correlated species. Previous studies have already shown that a merging of small electron and hole polarons into self-trapped excitons is unlikely^25^. In contrast, the question of whether STEs break up into pairs of oppositely-charged small polarons on a short time scale is still completely unsolved.

To address these points, an experimental approach with maximum temporal resolution has to be used. We therefore apply broadband femtosecond fluorescence upconversion spectroscopy, which provides luminescence spectra at fixed time delays with femtosecond temporal resolution^35^. For this purpose a very thin nonlinear optical crystal and a fixed non-collinear geometry is used for frequency mixing. Compared to the conventional upconversion scheme, where luminescence spectra are reconstructed from kinetic traces at single wavelengths, such an approach benefits from a faster measurement routine, a reduced background signal and a robust photometric correction procedure (see^35–37^ and this manuscript).

As an experimental novelty we adapt the existing scheme designed to measure molecules solved in liquids to solid samples. In detail, we combine a reflection geometry to collect luminescence known from the conventional upconversion scheme^38^ with the broadband non-collinear sum frequency mixing. This avoids deterioration of the temporal resolution introduced by the relatively large group velocity dispersion in lithium niobate. The apparatus response function has a full width at half maximum of $<160\,\hbox {fs}$ offering a temporal resolution much better than state of the art streak cameras^39^. The experimental setup is tested by measuring the reference system Coumarin 153 solved in dimethyl-sulfoxide (DMSO). The theoretical photometric correction procedure again proves to be very rigid^35^ and is further improved by additional consideration of the finite spectral bandwidth of the gate pulses. Both can be found in the supplementary information.

Samples with different stoichiometry and magnesium doping concentration are examined. First, it is demonstrated that it is possible to adapt such an experimental approach to the relatively weak room temperature luminescence of $\hbox {ABO}_3$ perovskite-like ferroelectrics such as lithium niobate. Since the broadband scheme naturally suffers from low conversion efficiencies, it is not clear a priori whether the luminescence intensity is sufficiently high for a detectable sum frequency signal. In a second step, the room temperature luminescence kinetics of a heavily Mg-doped LN crystal is compared with data at low temperature (50–200 K) measured with a photomultiplier and a gated photon counter. A combination of both experimental techniques extends the observed temperature range from 50 K to 300 K resulting in a much more reliable activation energy of the decay process compared to previous studies. Moreover, we are able to estimate an upper time limit for the ultrafast luminescence build-up and measure the luminescence decay of congruently melting lithium niobate. As these crystals exhibit the fastest and weakest luminescence decay of the LN compositions under study, the room temperature luminescence decay of samples with very different stoichiometries can be compared for the first time. Both luminescence formation and decay show a rather complicated temporal behavior, while no spectral change with time is observed. Whereas a second long, stretched-exponential decay component is clearly dependent on the defect structure, a fast exponential component with approximately 1 ps decay constant is present in each sample.

Since light-induced phenomena in LN are often related to the formation and transport of small polarons, which is usually studied by transient absorption spectroscopy, we additionally present fs-transient absorption data showing that luminescence and transient absorption decay times differ by an order of magnitude, which is in full agreement with Ref.^25^. The luminescence decay is largely independent of the small polaron absorption. Based on these findings, a microscopic picture is discussed that consistently describes the luminescence decay of the different samples. As microscopic origin self-trapped exciton decay and/or migration is suggested. Having shown elsewhere that it is unlikely that oppositely-charged small polarons merge into STEs^25^, our results indicate that the opposite case, i.e., STEs breaking into small polarons, does not occur either. It must therefore be concluded that self-trapped excitons and small polarons in lithium niobate are independent species.

## Results

### Transient luminescence of cLN:Mg (6.5 mol%)

**Figure 1:**
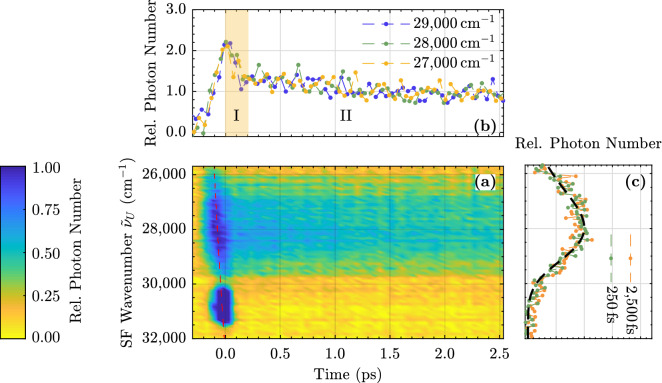
(**a**) Upconverted luminescence spectra of 6.5 mol% Mg-doped LN as a function of time up to 2.5 ps. The red dashed line illustrates the temporal delay between the luminescence kinetic traces at different wavenumbers introduced by their different group velocities in the long pass filter. (**b**) Normalized kinetic trace for three selected wavenumbers. (**c**) Detailed view of the spectra normalized to their maximum value for two fixed delay times (250 and 2,500 fs). The black dashed line is the steady state spectrum upconverted in silico (cf. blue curve in Fig. 5a).

Figure 1a shows the upconverted luminescence build-up of a congruently melting LN crystal with $6.5\,\hbox {mol}\%$ Mg in the melt. As the relative photon number decreases, the color coding changes linearly from blue to yellow. The upconverted spectra appear over a spectral range from $26,000$ to $30,000\,\hbox {cm}^{-1}$. A long pass filter in the optical path and a white glass filter in front of the optical fiber quench (upconverted) scattered pump light. Intensities above the maximum luminescence signal are cut off for better contrast. Therefore, the upconverted pump scatter appears as blue region without intensity information in the spectral range 30,000–$32,000\,\hbox {cm}^{-1}$ around time zero. A temporal change of the spectral shape and/or peak position cannot be deduced at a first glance. The small temporal delay between the luminescence maxima at different wavenumbers is due to different group velocities in the 1 mm thick fused silica long pass filter $\hbox {F}_1$ (red dashed line) and is easily corrected for further analysis in Fig. 1b.

**Figure 2:**
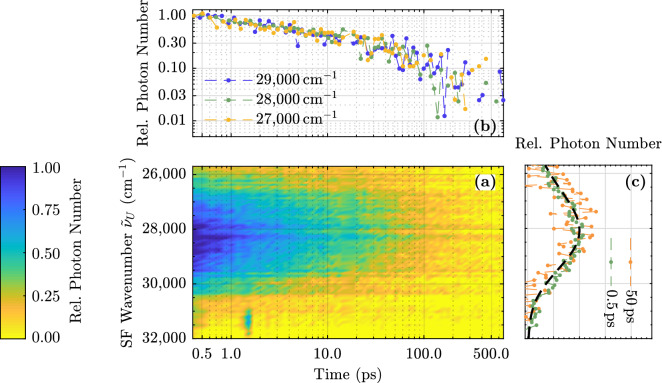
(**a**) Upconverted luminescence spectra of 6.5 mol% Mg-doped LN as a function of time up to 500 ps. (**b**) Normalized kinetic trace for three selected wavenumbers. (**c**) Detailed view of the spectra normalized to their maximum value for two fixed delay times (0.5 and 50 ps). The black dashed line is the steady state spectrum upconverted in silico (cf. blue curve in Fig. 5a).

Here, kinetic traces at different wavenumbers normalized to their value at 2.5 ps are shown for comparison. For each spectral component, a rapidly increasing photon number is observed on a 100 fs time scale, resulting in a luminescence peak near time zero, followed by a very fast and a slower decay component. The fast one, hereinafter referred to as decay I, is clearly visible within the first 200 fs, while the longer one (referred to as II) corresponds to the one shown in Fig. 2. Within the experimental error, the different traces exhibit identical temporal behavior, indicating that no temporal Stokes’ shift or broadening occurs within the first 2.5 ps.

A more detailed view of the upconverted spectra for different times is given in Fig. 1c. Obviously the spectral shape of the upconverted light does not change with time within the first 2.5 ps. For comparison, the steady state spectrum (see blue curve in Fig. 5a) is upconverted in silico to the expected distribution of UV photons and depicted in Fig. 1c as a dashed black line. The spectra agree almost perfectly.

The upconverted luminescence decay is shown in Fig. 2a. Now the white glass filter is removed, since the scattered pump light is not observed for later times. Accordingly, the upconverted spectra extend to higher wavenumbers. At first glance, no spectral change with time is observed here either. A detailed look at the decay kinetics of different detected wavenumbers supports this impression. All spectral components show the same non-exponential decay behavior (see Fig. 2b). Accordingly, the upconverted spectra do not change over time as shown in Fig. 2c up to 50 ps. Again, the steady state spectrum upconverted in silico is consistent with the measured data for all times (black dashed line).

**Figure 3:**
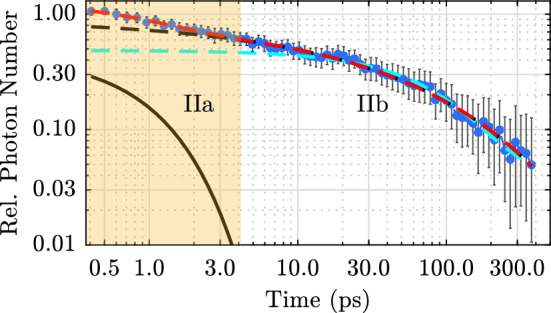
Normalized kinetic trace of the spectrally integrated luminescence of an Mg-doped (6.5 mol%) congruently melting sample. The turquoise dashed line is a fit of an exponential function to the data. The red dashed line is the sum of an exponential function (black straight line) and a Kohlrausch-Williams-Watts function (black dashed line) fitted to the data.

Since no spectral change is observed over time, further analysis is limited to the temporal evolution of the luminescence intensity. The spectra are spectrally integrated for each point in time to increase the signal-to-noise ratio. For this purpose, Gaussian functions are fitted to the distributions of UV photons and integrated over equally spaced wavenumber intervals. This suppresses the influence of pump scatter at short times on the total photon count. The result is shown in Fig. 3 as blue dots. The luminescence decays non-exponentially on the time scale of a few hundred picoseconds. For comparison, a fit of a single exponential function is shown as turquoise dashed line. A better fit to the data (dashed black line) is achieved applying a recently proposed microscopic model. There the temperature-dependent luminescence decay in lithium niobate is described by the local, radiative decay of self-trapped excitons and/or their migration and subsequent pinning at defect sites^25^. From this qualitative model we obtain, as explained in more detail in the discussion, a rate equation for the number density of transients *N*(*t*)

1 $$\begin{aligned} \frac{\partial N}{\partial t} = -\left( \tau _{\mathrm{r}}(T)^{-1}+\beta (T)\, t^{\beta (T)-1}\, \tau _{\mathrm{nr}}(T)^{-\beta (T)}\right) N(t,T), \end{aligned}$$

where $\tau _{\mathrm{r}}$ describes the local, radiative decay channel and $\beta \, t^{\beta -1}\, \tau _{\mathrm{nr}}^{-\beta }$ describes a stretched-exponential, non-radiative recombination path with the stretching factor $\beta$. This leads to the expression

2 $$\begin{aligned} N(t,T) = N_0(T)\,\text {exp}\left[ -\frac{t}{\tau _{\mathrm{r}}(T)}-\left( \frac{t}{\tau _{\mathrm{nr}}(T)}\right) ^{\beta (T)}\right] \end{aligned}$$

for the number density of transients as a function of time. The luminescence signal would then be

3 $$\begin{aligned} I(t,T) = p_{\mathrm{r}}(T)\, N(t,T) = N_0(T)\, \tau _{\mathrm{r}}(T)^{-1}\,\text {exp}\left[ -\frac{t}{\tau _{\mathrm{r}}(T)}-\left( \frac{t}{\tau _{\mathrm{nr}}(T)}\right) ^{\beta (T)}\right] . \end{aligned}$$

A special case of this function, i.e., with $\beta = 1/3$, has already been used to describe 1D diffusion of polaronic excitons to quenching centers in emissive conjugated polymers^40^. For elevated temperatures, at which the non-radiative decay channel dominates the relaxation process, equation (3) can be approximated by a simple Kohlrausch-Williams-Watts function ($\propto \mathrm {exp}(-(t/\tau )^{\beta })$). The region IIb in Fig. 3 can be fitted with such a function with a non-radiative decay time $\tau _{\mathrm{nr}} = (31\pm 13)\,\hbox {ps}$ and a stretching factor $\beta = 0.43\pm 0.06$ (black dashed line). For an overall fit of the decay kinetics (red dashed line), an additional exponential component (straight black line) is required to reflect the deviation between the data and the fit function equation (3) in region IIa. Its decay time is 1 ps. Based on the microscopic model used, we therefore obtain two decay components in the temporal region II. Together with the ultrafast decay I a three-component decay is observed.

**Figure 4:**
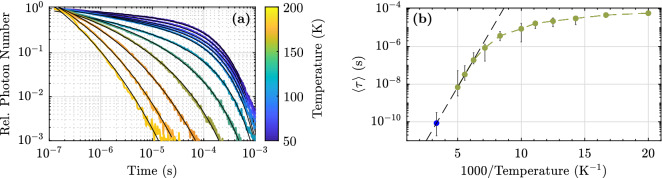
(**a**) Luminescence decay of a heavily Mg-doped LN crystal (6.5 mol% Mg) for different temperatures between 50 and 200 K, obtained with a photomultiplier and a gated photon counter at an emission wavelength of 440 nm. The kinetic traces are fitted with equation (3) (black lines). (**b**) Luminescence mean decay time calculated via equation (4) as a function of temperature. The green dots are calculated from fits of the left part of this figure. The blue dot is the mean decay time (IIb) obtained via upconversion spectroscopy. The black dashed line is an Arrhenius fit.

For comparison, the luminescence decay at low temperatures at a single wavelength of 440 nm is measured with a photomultiplier and a gated photon counter. Again fs-laser pulses at 400 nm are used to excite the sample. The experimental setup, but using ns-pump pulses, has already been described in Ref.^25^. The luminescence decay kinetics for temperatures between 50 K and 200 K are fitted with equation (3) and depicted in Fig. 4a. The radiative decay time can be determined for temperatures up to 120 K and has a value independent of temperature of $\approx 220\,\mu \hbox {s}$. Above this temperature $\tau _{\mathrm{nr}}\ll \tau _{\mathrm{r}}$ and $\tau _{\mathrm{r}}$ can be neglected. The mean decay time $\langle \tau \rangle$ is calculated for every temperature via

4 $$\begin{aligned} \langle \tau \rangle (T) = \frac{1}{N_0(T)}\, \int _0^{\infty } N(t,T)\text {dt} \end{aligned}$$

and is depicted in Fig. 4b, where the mean decay time (IIb) at room temperature is depicted as a blue dot. For temperatures $\ge 160\,\hbox {K}$ an Arrhenius function is fitted to the data (black dashed line). The resulting activation energy is $E_{\mathrm{a}} = (0.23\pm 0.04)\,\hbox {eV}$.

### Composition-dependent kinetics

**Figure 5:**
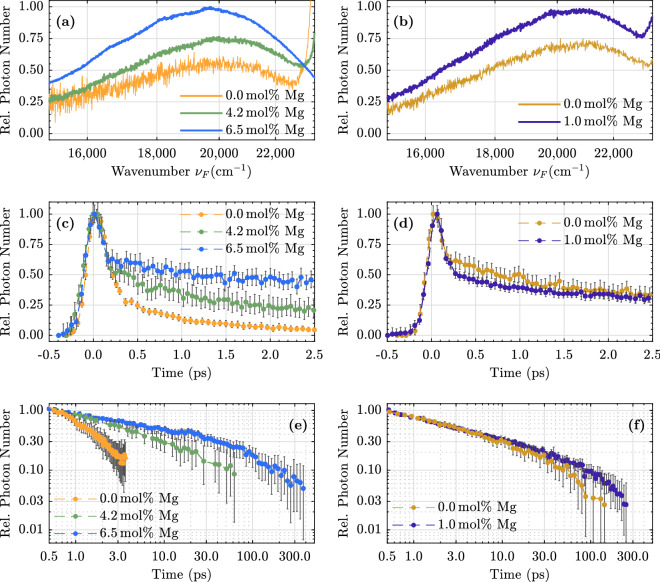
Steady state luminescence spectra (**a**) and (**b**), normalized short-time (**c**) and (**d**) and long-time kinetic traces (**e**) and (**f**) for lithium niobate samples with different stoichiometries/Mg-dopant concentrations. **Left column:** congruently melting crystals without Mg-doping (yellow), $4.2\,\hbox {mol}\%$ Mg (<ODRT, green) and 6.5 mol% Mg in the melt (>ODRT, blue). **Right column:** near-stoichiometric samples without Mg-doping (dark yellow) and 1 mol% Mg (>ODRT, dark blue).

Similar investigations at room temperature were performed for samples with different stoichiometries/Mg-dopings. The results are presented in the Supplementary Information. All of them have in common that no spectral change is observed over time. Further analysis is therefore again limited to the luminescence intensity. The result is shown in Fig. 5, in which the luminescence build-up and decay of congruently melting samples (left column, (c)/(e)) and near-stoichiometric samples (right column, (d)/(f)) with different Mg-doping concentrations are compared. All samples show the before-mentioned intensity peak around time zero. Various measurements indicate that this peak is related to surface degradation, as it seems to become more pronounced the longer a sample is illuminated.

However, the luminescence kinetics of the different samples reveal a further correlation. Each luminescence signal again consists of two components, a short exponential decay IIa with a decay time of $\tau = (1\pm 0.5)\,\hbox {ps}$ and a longer non-exponential decay IIb with a stoichiometry-dependent decay time.

While in the congruent sample (yellow dots) the exponential decay dominates the overall signal, successive Mg-doping (green and blue dots) lead to an increased contribution of the longer non-exponential component. The change from congruently melting compositions to near-stoichiometric samples shows, that this long component is also present without any Mg-doping (dark yellow dots). A magnesium-doping above the optical damage resistance threshold (1 mol% Mg in the melt) of such samples prolongs the luminescence decay only slightly (dark blue dots). Sorting the samples by the length of the longer decay and the ratio of the amplitude of the short and long decay leads to the same order.

### Transient absorption

As a complementary experimental technique, transient absorption spectroscopy is performed to probe the light-induced transients. Again, the intense laser pulses at 400 nm are used as pump source. Probe pulses with wavelengths of 910 nm and 460 nm are provided by the optical parametric amplifier used in the upconversion setup. Both pulses are focused on the samples with concave mirrors (f = 500 mm) at a small angle of $\approx 6^{\circ }$. The probe light is ordinarily polarized with respect to the optical axes of the lithium niobate samples. The pump pulses are blocked, while the probe pulses are focused on the entrance of the optical fiber connected to the same spectrograph as in the fluorescence upconversion setup. Spectral integration of the probe pulses for each time step *I*(*t*) is used to calculate the absorbance

5 $$\begin{aligned} A(t) = -\mathrm {log}_{10}\left( \frac{I(t)}{I(t\ll 0)}\right) , \end{aligned}$$

where $I(t\ll 0)$ is the spectrally integrated pulse signal for time delays, where the probe pulse arrives at the samples before the pump pulse. The apparatus response function has a full width at half maximum of 130 fs.

**Figure 6:**
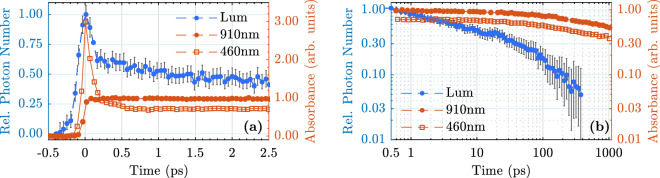
Transient absorbance of cLN:Mg (6.5 mol%) measured at probe wavelengths 910 nm and 460 nm (red dots and squares, respectively) compared with the transient spectrally integrated luminescence (blue dots). (**a**) Short-time behavior up to 2.5 ps, (**b**) Longer decay up to 1 ns.

Figure 6 shows the ultrafast transient absorbance of the cLN:Mg sample with a Mg concentration above the optical damage resistance threshold probed at 910 nm and 460 nm, corresponding to absorption bands typically associated with electron and hole polarons in lithium niobate. First, no short-time exponential decay is observed for the two wavelengths (Fig. 6a, red dots and squares). Figure 6b shows that the transient absorption is considerably longer than the luminescence decay (blue dots). On a time scale of a about 10 ps, it is more or less constant. This measurement is performed for the other samples as well. The results qualitatively confirm once again, that no absorption feature corresponding the luminescence decay is observed in the blue-green and near infrared spectral range.

## Discussion

For the first time, broadband femtosecond fluorescence upconversion spectroscopy has been successfully applied to the photoluminescence of lithium niobate single crystals. To the best of our knowledge, it is the first measurement of this kind on $\hbox {ABO}_3$ perovskite-like ferroelectrics. In comparison to former studies^24–26,31–34^ this experimental approach uncloses the detection range for femtosecond/picosecond luminescence kinetics. In particular, it unlocks the room temperature sub-ns temporal behavior of lithium niobate single crystals with different stoichiometry and Mg-doping concentrations. Even congruently melting samples known as weak emitters can be examined. Their luminescence decay time has so far not been satisfactorily determined even at low temperatures^32^. We are therefore convinced that broadband fluorescence upconversion spectroscopy can also be applied on other (weakly) luminescing dielectrics with strong electron-phonon coupling of current interest, such as $\hbox {TiO}_2$ or $\hbox {SrTiO}_3$ and may provide new insights into carrier decay channels.

The necessity to use a broadband upconversion scheme arises as a potential time-dependent Stoke’s shift of the luminescence feature could result in misleading kinetic traces observed for a single wavenumber. This experimental approach is preferred, e.g., to a streak camera, because in principle a much better temporal resolution can be achieved and a comparison to pump-probe transient absorption measurements on the fs-time scale might be easier.

In a first step we have shown that within the experimental error no temporal change of the luminescence band, which e.g. indicates a relaxation of hot carriers to an intermediate ground state, is observed (see Figs. 1 and 2). We like to remind that our experimental setup, although designed as a broadband scheme, has a limited access to the spectral shape of a very broad luminescence band. The steady state luminescence spectra exceed the spectral bandwidth (FWHM) of our setup by about a factor of two. Calculations showed that the peak position of such a broad spectrum can be determined with an error of $\pm 400\,\hbox {cm}^{-1}$. This means that two very broad emission bands shifted by $\pm 50\,\hbox {meV}$ are difficult to distinguish within the current signal-to-noise ratio. Therefore, further analysis is limited to the temporal evolution of the luminescence intensity.

The observed pronounced luminescence maximum around time zero, which decays on the fs time scale (cf. Fig. 5c and d) is a rather surprising result, since it has the same spectral fingerprint as the longer decay components (cf. Fig. 1). Thus, upconverted scattered pump light at the origin of this decay, which could be the first assumption, is unlikely. In this case the influence on the observed kinetics would decrease with decreasing wavenumber. Furthermore, a closer examination reveals that the intensity peak is temporally broader than the cross correlation signal (FWHM 210 fs vs. $\le 160\,\hbox {fs}$). Moreover, we like to point out that the low-energy part of the detected upconverted luminescence signal corresponds to wavelengths in the region of 540 nm, which is spectrally far away from a Gaussian-shaped laser pulse centered at 400 nm having a bandwidth of a few nm, even if a potential low energy shoulder is considered. However, we had the impression that the intensity ratio between the peak and the ps decay increases the longer the sample is illuminated. Therefore, we cannot exclude that the peak is related to surface degradation. Although, as we have argued above, it is rather unlikely that scattered pump light is responsible for this feature, we will postpone the discussion about the peak to later times. In the current experiment, the upconverted luminescence signal near time zero is ’caged’ between pump scatter on the low energy side and upconverted pump scatter on the high energy side. Further experiments with a different pump wavelength, e.g., frequency-tripled laser pulses at 266 nm, might therefore clarify the luminescence behavior on the fs time scale, since in this case a spectral shoulder of the upconverted scattered pump light could definitely be excluded.

### Luminescence features

In a more detailed discussion we focus on the two remaining ps-decay components IIa and IIb (cf. Fig. 3). Regarding its luminescence kinetics, congruently melting lithium niobate doped with Mg above the optical damage resistance threshold is a frequently investigated crystal composition^24,25,32,33^. We therefore begin our comparison to results reported in the literature with this sample composition. First, we present here the first fs-pulse induced data on luminescence kinetics at low temperatures (50 K – 200 K, cf. Fig. 4). They are in accordance with ns-pulse induced kinetics presented in the literature^24,25^, which proves that the decay time, at least at lower temperatures, is not influenced by the pump pulse duration and intensity, which in our case is more than five orders of magnitude shorter and three order of magnitude higher, respectively. For a quantitative comparison of the decay shape, we determined $\tau$ and $\beta$ using the previously applied first derivative of a stretched-exponential function^24,25^ (not shown) resulting in the same fitting parameters for fs- and ns-pulse-induced luminescence kinetics.

However, such approach is based on the assumption that only a radiative decay channel is present, which is not reasonable for elevated temperatures. We therefore use an expression according to equation (3) as fitting function to compare the luminescence kinetics at low temperature and at room temperature. It is based on a recently proposed model that describes the low-temperature luminescence/absorption decay in lithium niobate considering STEs^25^. In particular, it is assumed that STEs have two decay channels, one local and radiative and one non-local, non-radiative, respectively. The latter describes STE migration through the crystal and pinning at defect sites. The complexes formed decay non-radiatively. As shown in Fig. 4a an excellent fit quality can be achieved. The radiative decay time turns out to be independent of temperature. The mean decay times as a function of temperature are shown in Fig. 4b. The inclusion of the mean decay time obtained from upconversion spectroscopy at room temperature (blue dot) in an Arrhenius fit leads to an activation energy of $E_{\mathrm{a}} = (0.23\pm 0.04)\,\hbox {eV}$, which is larger than a recently published value obtained from low-temperature measurements on a sample with an Mg cocentration of $7\,\hbox {mol}\%$ in the melt ($E_{\mathrm{a}} = (0.14\pm 0.01)\,\hbox {eV}$)^24^. The reason for this is the extended temperature range in our experiment. Figure 4b shows that for temperatures below 160 K the mean decay time becomes increasingly temperature-independent. Since an Arrhenius function leads to an excellent fit of the temperature-dependent decay time, it is likely that the longer decay IIb at room temperature reported here corresponds to the commonly investigated $\mu$s luminescence decay in Mg-doped lithium niobate at low temperatures.

A change of the crystal composition, i.e., the stoichiometry and/or Mg-dopant concentration, largely influences the decay time of the longer component IIb as shown in Fig. 5e and f. The near-stoichiometric sample proves that the occurrence of this decay component is not related with Mg-dopants in the sample. The near-stoichiometric samples with and without Mg-doping have comparable decay times. The slightly shorter decay time of the undoped sample could be caused by impurity quenching due to residual antisite defects. This hypothesis is supported by the fact that the congruently melting sample with a Mg-doping just below the ODRT (4.2 mol%) has a very comparable decay time. In contrast, the congruently melting samples differ significantly from one another. With decreasing Mg concentration, the longer decay time decreases and the ratio between the short exponential (IIa) and the longer non-exponential component (IIb) at 1 ps after excitation increases. Since Mg-ions at the origin of this decay can be excluded, it seems likely that antisite defects, whose number density is anti-proportional to the Mg concentration, efficiently quench this long decay component. Anyhow, regardless of a concrete microscopic picture, the decay channel is considered to be of non-radiative nature, since the collected photon number of the longer component 1 ps after excitation is constant in all samples.

**Figure 7:**
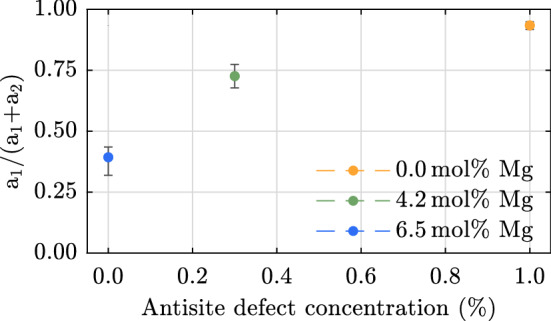
Amplitude ratio of the exponential ($a_1$) and non-exponential ($a_2$) decay in congruently melting lithium niobate samples as a function of antisite defect concentration. For short times the non-exponential decay is approximated by an offset. The antisite defect concentration for the samples with 4.2 mol% Mg in the melt is estimated from the results given in Ref.^41^.

An even shorter decay component, corresponding to the exponential decay (IIa) in this work, was not observed by the two authors mentioned above. In contrast, Pankratov *et al.* reported a similar phenomenon in stoichiometric lithium niobate. They found a two-component decay over the entire luminescence spectrum. At liquid nitrogen temperatures, the two decay times were estimated to be $<20\,\hbox {ns}$ and $2\,\mu \hbox {s}$, respectively^31^. Fischer *et al.* observed a second, very fast component in Zn-doped LN samples as well^32^. At the origin of this faster component, both suspected residual antisite defects in their samples, which are expected to accelerate the luminescence decay^26^. However, a systematic study of samples with different compositions was not performed by these authors. The data given in Fig. 5c and d also shows that the ratio between the short exponential decay and the longer non-exponential decay depends largely on the stoichiometry of the crystals. The higher the concentration of antisite defects, the more pronounced the short component appears. Figure 7 shows the amplitude ratio between the exponential decay component ($a_1$) and the longer one as a function of antisite defect concentration. For the very short time considered, the latter can be approximated as constant ($a_2$). The reason for the approximation is that a stretched-exponential function diverges for small arguments, which leads to unphysical results at very short times. The antisite defect concentration for the sample with 4.2 mol% Mg in the melt is estimated from the results given in Ref.^41^. However, the number of photons collected from the exponential decay channel is more or less the same in the three congruently melting samples, regardless of the Mg-doping. Since the number density of antisite defects and lithium vacancies varies greatly between these samples, we rather assume that the short exponential decay is related to the regular Nb-O-octahedra.

### Microscopic picture

Light-induced phenomena in lithium niobate are often related to formation and transport of small polarons. A microscopic model describing transient photoluminescence data should therefore naturally take small polaron formation and decay into account. Since small polaron kinetics are usually studied by means of transient absorption spectroscopy, we report here on room temperature transient absorption data obtained under the same experimental condititons as the luminescence data. The transient absorption build-up and decay at a probe wavelength of 910 nm are in accordance with previously reported signals probed at 800 nm and in the NIR spectral range^19,21,23^. Both probe wavelengths reported here (910 nm and 460 nm) are time-locked probing the same decay mechanism, i.e., geminate electron and hole polaron annihilation. The sharp peak observed around time zero in the blue spectral range might be caused by two-photon absorption. A comparison with the luminescence decay (cf. Fig. 6), again clearly shows no correlation with the transient absorption kinetics, neither for the short exponential decay IIa nor for the longer non-exponential component IIb. In particular, the large difference in the decay times at room temperature of one order of magnitude suggests that the luminescence decay kinetics on the ps-time scale is not influenced by small polaron absorption.

In the literature there are further indications leading to the conclusion that the blue-green photoluminescence in lithium niobate is not caused by geminate small polaron annihilation or radiative small polaron decay^18,25^. Instead, Messerschmidt *et al.* recently revived the idea of self-trapped excitons located at the Nb-O-octahedra at the origin of the luminescence^9,25–28^, which has been supported by ESR studies indicating, that the X-ray induced luminescence is associated with electrons captured at $\hbox {Nb}^{5+}$ and holes captured at $\hbox {O}^{-}$ ions^42^.

A possible explanation for a two component luminescence decay might then be found in two STE species with nearly identical spectroscopic fingerprints, which are directly generated by the pump pulse. Various kinds of intrinsic STEs are known in the literature, which differ in their location, e.g., on-center or off-center, or their multiplicity (see^43^ and references therein). The latter can lead, for example, to singlet and triplet emission bands separated by a few tens of meV (rare gas solids) or a few eV (alkali halides). Typically, singlet and triplet STEs decay on very different time scales. For instance, triplet STE decay in rare gas solids is two to four orders of magnitude slower, depending on the temperature. In the wide-gap oxide $\hbox {Al}_2\hbox {O}_3$ very different decay times for singlet and triplet self-trapped excitons are found as well^44^.

Transferred to the perovskite-like ferroelectric lithium niobate, the often observed luminescence of the intrinsic niobium-oxygen octahedra in LN might then be assigned to a triplet state, since it decays at cryogenic temperatures within a few hundred $\mu \hbox {s}$^25,26^, which is a typical time scale for triplet STE decay (see^43^ and references therein). In fact, photo-EPR studies on $\hbox {KTa}_{1-x}\hbox {Nb}_x\hbox {O}_3$ revealed the existence of triplet $\hbox {Nb}^{4+}-\hbox {O}^{-}$ polaronic excitons^45^. They share a common electronic structure with charge transfer vibronic excitons $\hbox {Ta}^{4+}-\hbox {O}^-$ and $\hbox {Ti}^{3+}-\hbox {O}^-$, whose triplet configurations are assumed to be their ground states and which are related with green luminescence in $\hbox {KTaO}_3$ and $\hbox {SrTiO}_3$, respectively^46–48^. Calculations furthermore revealed that the recombination of triplet charge transfer vibronic excitons at the niobium oxygen octahedra in $\hbox {KNbO}_3$ would lead to the experimentally observed blue/green luminescence as well^48,49^. In our samples, triplet STEs might survive long enough to migrate through the crystal until getting trapped/pinned at defect sites, which efficiently quenches the luminescence^25^. A distribution of distances to these defect sites would then manifest in a stretched-exponential decay behavior and the number density of defect centers influences the mean decay time. A high defect number density increases the pinning probability, resulting in shorter decay times, which would be consistent with our findings. A fitting function based on this idea (cf. equation (3)) leads to an excellent fit quality for very different temperatures. The additional short exponential decay component at room temperature might be attributed to locally decaying singlet STEs that cannot hop through the crystal lattice due to a very short intrinsic decay time.

From Fig. 5c and d it is immediately clear that the formation time of these quasi-particles is $\le 200\,\hbox {fs}$. Such a time scale for STE formation is remarkably short and has been observed in polydiacetylen and $\hbox {SiO}_2$ as well^50–53^. At low temperatures, time constants on the ps time scale are often reported, e.g., in alkali halides^54,55^, where self-trapping of excitons is hindered by a small energy barrier introduced by the short range coupling to acoustic phonons. Since our study is conducted at room temperature, such an energy barrier might be negligible, since typically only a few meV have to be surpassed^56^. However, this formation time is in the same range as the recently reported values for small polaron formation, which were estimated to lie between 1.5 fs and 300 fs^21–23^. As on the very short time scale the luminescence decay is again not accompanied by an increase of the transient absorption signal, this suggests that STEs do not break up into pairs of small polarons. Of course, the net absorption cross section of the small polaron pair could coincidentally correspond to that of the STE. But since this would be an unlikely accident, our investigation again indicates that both species are independent of each other.

Time-dependent density functional theory calculations might provide further insight into the microscopic processes responsible for the ultrafast luminescence decay behavior reported here and could help to verify whether our proposed model is justified.

In summary, this study reveals the sub-ns temporal dynamics of photoluminescence of the $\hbox {ABO}_3$ perovskite-like ferroelectric lithium niobate with ultrafast temporal resolution. To this end, it is shown that broadband fluorescence upconversion spectroscopy is feasible on such materials, although their luminescence is efficiently quenched at elevated temperatures. Within the error of our experiment, no temporal Stoke’s shift is observed. Samples with different stoichiometries and Mg-dopant concentrations show a different long time behavior, which manifests itself in non-exponential decay kinetics. A larger number of antisite defects in the samples correlates with an efficient quenching of this longer component. On the other hand, a shorter exponential decay component is present regardless of the crystals composition.

Fs-transient absorption spectroscopy data on the same samples are qualitatively in accordance with already published kinetics and show that geminate small polaron annihilation can be excluded as microscopic mechanism for the blue-green luminescence of lithium niobate. Instead, a model based on self-trapped excitons is proposed. Both species seem to be independent of each other. For a complete and rigid microscopic picture, however, further experimental and theoretical investigations are required.

## Methods

### Experimental setup

Picosecond transient luminsecence kinetics of lithium niobate samples is investigated using broadband fluorescence upconversion spectroscopy (FLUPS)^35–37^ adapted to the inspection of single crystals in reflection geometry. The samples are excited with frequency-doubled fs laser pulses (Coherent Inc. type *Astrella*, $\lambda = 400\,\hbox {nm}$, $\tau = 60\,\hbox {fs}$, $E = 65\,\mu \hbox {J}$) at a repetition rate of 1 kHz. A Cassegrain reflector collects the emitted fluorescence and images the fluorescing spot on a BBO crystal (Altechna, $\hbox {d} = 130\,\mu \hbox {m}$, $\theta _{\mathrm{N}} = 40\,^\circ$, $\phi = 0\,^\circ$, front p-coating @ 400–1300 nm, backside p-coating @ 300–450 nm). There it is mixed under non-collinear Type II phase matching condition with NIR-gate pulses ($\lambda = 1340\,\hbox {nm}$, $\tau = 45\,\hbox {fs}$, $E = 50\,\mu \hbox {J}$). In order to compensate for pulse front mismatch introduced by the large mixing angle $\alpha \approx 19^{\circ }$, the gate pulse fronts are tilted by $\approx 20\, ^{\circ }$ by means of an equilateral N-SF11 prism and a demagnifying imaging of the spatial pulse profile on the BBO crystal. While the luminescence and gate pulses are blocked, the generated sum-frequency signal is imaged onto the entrance of an optical fiber connected to a spectrograph (Roper Scientific, type *IsoPlane* and *PIXIS 2K*). The sum-frequency signal of scattered pump light defines time zero and yields the temporal apparatus response function, which has a Gaussian shape with a full width at half maximum of $<160\,\hbox {fs}$. For each fixed time delay, the upconverted spectra are integrated for 5 s and averaged over 10 measurements (median), i.e., over 5,000 pump events.

A detailed description of the experimental setup as well as test measurements on the reference system Coumarin 153 solved in dymethyl-sulfoxide and a photometric correction, which extends the procedure established for a similar setup, are included in the supplementary information.

### Samples

Room temperature luminescence kinetics (ordinarily polarized part) and transient absorption are studied on plates of lithium niobate with different stoichiometries and Mg-doping concentrations.

The congruently melting samples (cLN) were grown by the Czochralski method from sintered mixtures of $\hbox {Li}_2\hbox {CO}_3$ and $\hbox {Nb}_2\hbox {O}_5$ with a Li/Nb molar ratio of 0.946 corresponding to the congruent composition. For doping, MgO was added to the starting mixture to obtain the specific molar Mg ratio in the melt ($4.2\,\hbox {mol}\%$ and $6.5\,\hbox {mol}\%$, respectively).

The undoped and Mg-doped stoichiometric samples (sLN) were grown by the high-temperature top-seeded solution growth (HTTSSG) method using $\hbox {K}_2\hbox {O}$ flux of a concentration of $\approx 13\,\hbox {mol}\%$ (see^3^) from (doped) starting mixtures with the stoichiometric Li/Nb molar ratio. All crystals were grown, monodomenized close to the melting temperature, cut, and polished to optical quality at the WIGNER Research Centre for Physics, Budapest.

## Supplementary information

Supplementary Information.

## Data Availability

The data that support the findings of this study are available from the corresponding author upon reasonable request.
