# Supplementary material for: Superposed picosecond luminescence kinetics in lithium niobate revealed by means of broadband fs-fluorescence upconversion spectroscopy
Source: Sci Rep. 2020 Jul 9;10:11397. doi: 10.1038/s41598-020-68376-6 (PMC7347870; doi:10.1038/s41598-020-68376-6)
Supplement: Supplementary file 1 — Supplementary Information. [file 41598_2020_68376_MOESM1_ESM.pdf]

Superposed picosecond luminescence kinetics in lithium niobate revealed by means of broadband fs-fluorescence upconversion spectroscopy

- Supplementary Information -

A. Krampf[1], S. Messerschmidt[1], and M. Imlau*[1]

[1]School of Physics, Osnabrueck University, Barbarastraße 7, 49076 Osnabrueck, Germany
*mirco.imlau@uni-osnabrueck.de

Reflective broadband fluorescence upconversion spectrometer

Experimental Setup

The experimental setup for the investigation of the luminescence kinetics of solid samples (cf. Fig. S 1) follows the idea of a broadband upconversion scheme introduced by Ernsting *et al.* for the measurement of the luminescence of solved molecules[1]. A regeneratively amplified Ti-Sapphire laser (λ = 800 nm, τ = 35 fs, Coherent Inc. type *Astrella*) delivers intense, frequency-doubled laser pulses (λ = 400 nm, τ = 60 fs, E = 65 μJ) at a repetition rate of 1 kHz. After passing a motorized linear translation stage (travel distance of 300 mm), they are focused on the sample by a concave mirror (M_1, f = 250 mm).

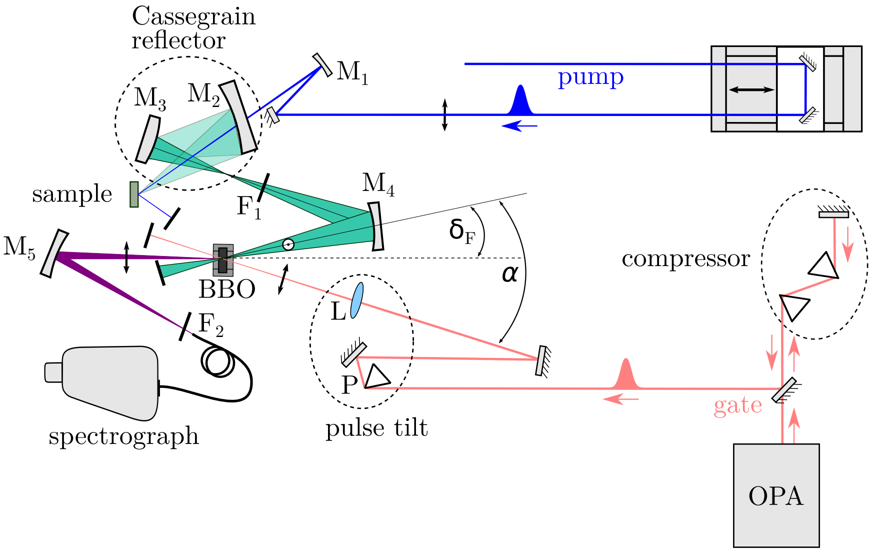

Figure S 1. Experimental setup used for broadband fluorescence upconversion spectroscopy of single crystals in reflection geometry. The sample is excited with a fs-laser pulse at a wavelength of 400 nm. The emitted luminescence is collected and imaged by an off-axis Cassegrain configuration onto a thin BBO crystal. There it is mixed with compressed and tilted gate pulses. The central ray of the luminescence cone and the gate pulses enclose an angle of $\alpha \approx 19°$. The generated sum frequency is imaged onto the input of an optical fiber and analyzed by a spectrograph. **F**: filter, **M**: spherical mirror, **L**: lens, **P**: prism.

The emitted luminescence is collected with a pair of spherical mirrors forming an off-axis Cassegrain reflector (M_2, R = -150 mm and M_3, R = 258.4 mm, magnification ≈3). It is designed to reduce astigmatism. The effects of such aberrations on the time resolution of our setup are negligible, which is checked considering ray optics. In contrast to the reference setup[1], the imaging of the fluorescent spot is realized in a reflective configuration. For this purpose the first, larger mirror of the Cassegrain reflector has a hole drilled in its center. Since two photon absorption decreases strongly with increasing penetration depth, the induced luminescence is predominantly emitted from a very thin subsurface layer. This geometry thus reduces a deterioration of the temporal resolution introduced by the relatively large group velocity dispersion in the relevant blue/green spectral range. While the specularly reflected pump light is blocked with a beam dump, pump light scattered from the sample surface is suppressed by a longpass filter with a cut-on wavelength of 422 nm (F_1, Asahi Spectra USA Inc. *ZUL0422*). Another concave mirror (M_4, R = -300 mm) then re-images the magnified image of the fluorescent spot without further magnification onto a BBO crystal with anti-reflection coatings on its front and backside (Altechna, d = 130 μm, θ_N = 40°, ϕ = 0°, front p-coating @ 400–1300 nm, backside p-coating @ 300–450 nm).

The luminescence is mixed under non-collinear Type II phase matching condition with NIR-gate-pulses (λ = 1340 nm, τ = 45 fs, E = 50 μJ) generated by an optical parametric amplifier (Light Conversion type *TOPAS Prime*). Since we expect a very weak luminescence signal, we use the more efficient BBO orientation called "Case A" in Ref.[1], which converts a broad cone of incident light via angular tolerance phase matching. The large angle between the luminescence and the gate pulse ($\alpha \approx 19°$) naturally deteriorates the temporal resolution of the experiment for geometric reasons. To reduce this effect, the gate pulse fronts are tilted by ≈ 3.2° using an equilateral N-SF11 prism (P). The tilt angle is calculated using the description in Ref.[2]. A convex lens (L, f = 100 mm) on a dovetail translation stage images the gate pulses onto the BBO crystal with a demagnification of ≈ 6.5, stretching the tilt angle to ≈ 20°. A prism-compressor compensates for the pulse broadening introduced by the group velocity dispersion in the N-SF11 prism.

The non-collinear wave mixing allows a spatial filtering of the generated from the fundamental waves. While the luminescence and gate signals are blocked, an UV-reflectance enhanced concave aluminum mirror (M_5, R = -200 mm) images

the sum-frequency with a small demagnification onto an optical fiber connected to a spectrograph with thermoelectrically cooled CCD (Roper Scientific, type *IsoPlane* and *PIXIS 2K*). With an additional long pass filter (F_2, Schott *WG335*) in front of the fiber, the sum-frequency signal of scattered pump light can be reduced around time zero if necessary. This signal defines time zero in the experiment as well as the apparatus response function, which has a Gaussian shape with a full width at half maximum of <160 fs.

The spectral region of efficient upconversion in a non-collinear fluorescence upconversion scheme depends largely on several parameters such as gate pulse wavelength, angles of incidence and BBO crystal thickness. Tuning the fluorescence angle δ_F by only a few degrees shifts this spectral region over the entire visible spectrum[1]. Therefore, before a measurement can be performed, spectral efficiency curves are calculated to find the fluorescence angle that provides the desired spectral region. To measure the luminescence kinetics of the lithium niobate samples, the fluorescence angle is set by turning the BBO crystal mounted on a calibrated, motorized rotation stage, so that the corresponding region of efficient upconversion is close to the room temperature steady state luminescence maximum near $\approx 20{,}000\,\mathrm{cm}^{-1}$ (cf. Fig. 5 (a) and (b) in the manuscript). For each fixed time delay, the upconverted spectra are integrated for 5 s and averaged over 10 measurements (median), i.e., over 5,000 pump events.

Photometric Correction

Recently, Gerecke *et al.* compared photometric correction curves for their broadband fluorescence upconversion spectroscopy setup, which were obtained from upconverting different fluorescing dyes and SFG efficiency calculations, respectively[1]. For their efficiency curve generated in silico, they essentially considered the non-collinear geometry, i.e., the angles of the incident fluorescence and gate pulses, and the fluorescence divergence resulting from the large collection angle and the magnifying imaging on the nonlinear optical crystal. Including a single gate wavelength in their calculations, which reflects the central wavelength of the gate pulses, leads to a good agreement between both correction curves, especially in the wavelength region of (nearly) perfect phasematching. In a more recent study, however, they showed that relatively small changes in the gate wavelength have a large impact on the efficiency (correction) curve, showing that an accurate determination of the gate wavelength is imperative[3].

Therefore, we extend the calculation by including the spectral shape of the gate pulses, which is determined beforehand having a finite bandwidth of ≈ 50 nm (full width at half maximum). The spectral parts are taken into account according to their fraction of the total gate pulse energy. This naturally broadens the region of efficient sum frequency generation, since each spectral part of the gate pulse fulfills the condition of perfect phase matching for a different luminescence wavelength. The difference between finite and arbitrarily small gate bandwidth is illustrated in Fig. S 2. The same experimental parameters regarding crystal properties and geometry are used as in our measurement. The fluorescence angle is set to $\delta_F = 2.5°$, which is later used for the measurement of lithium niobate samples. The dashed blue line results when only a single wavelength at 1340 nm is considered. A maximum conversion efficiency is obtained for $\tilde{\nu}_\mathrm{F} \approx 20{,}600\,\mathrm{cm}^{-1}$ and an efficiency of more than 30% of the maximum value in the range of $18{,}060\,\mathrm{cm}^{-1}$–$22{,}350\,\mathrm{cm}^{-1}$. The more realistic efficiency including the actual bandwidth of ≈ 50 nm of the gate pulses has its maximum again at $\tilde{\nu}_\mathrm{F} \approx 20{,}600\,\mathrm{cm}^{-1}$, but is broadened (cf. orange line in Fig. S 2). If we again take a value of 30% of the maximum as a reference, the region of efficient upconversion is broadened by $670\,\mathrm{cm}^{-1}$. Conversely, the correction curve assuming only a single wavelength is narrower, which leads to an overestimation of the fluorescence bandwidth. It should be noted that the absolute effect of the finite gate pulse bandwidth depends largely on the crystal thickness and the fluorescence angle. It is increased with thicker crystals and larger fluorescence angles.

Coumarin 153 solved in dimethyl-sulfoxide

To validate that our setup works as expected, we measured the luminescence of Coumarin 153 (*Aldrich*, CAS: 53518-18-6) solved in dimethyl-sulfoxide diluted to optical density ≤ 0.3 at 400 nm using our reflection geometry and compared the results with the steady state spectrum and literature. The filled volume of the cuvette is placed in the object plane of the Cassegrain reflector. In a first step, the measured spectra are corrected for the spectral response of the fiber and spectrograph determined with a standard light source (Bentham *CAL_CL2_100W*). The measured relative photon numbers $s(\lambda) = s(\lambda,t) - s(\lambda, t \ll t = 0)$ are converted to the energy scale according to the Jacobian transformation

$$s(\tilde{\nu}) = s(\lambda)\frac{\mathrm{d}\lambda}{\mathrm{d}\tilde{\nu}} = -s(\lambda)\,\lambda^2.$$

Figure S 3 shows the measured instantaneous upconverted fluorescence spectra of Coumarin 153 solved in DMSO 1 ps and 500 ps after excitation, recorded with our setup as blue and yellow dots, respectively. The fluorescence angle is set to $\delta_F = 3.5°$. The angle α is fixed.

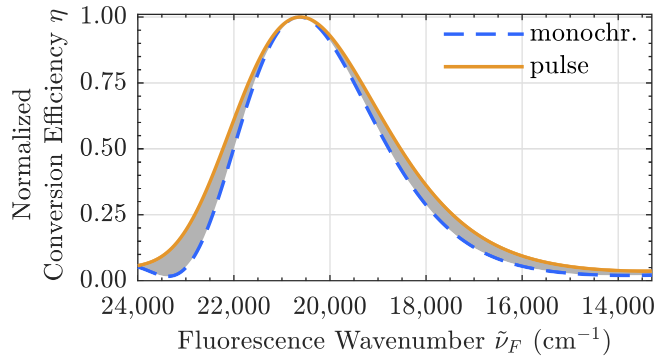

Figure S 2. Normalized spectral conversion efficiency of a thin BBO crystal (d = 130 μm, θ_{N} = 40°, ϕ = 0°) set to δ_F = 2.5° with (orange) and without (dashed blue) consideration of the gate pulse bandwidth of $\approx$ 50 nm (central wavelength 1340 nm). Both are normalized to their respective maximum. The external angle between fluorescence and gate pulse is $\alpha \approx 19°$. Type II phase matching is used.

To fit these data, a (test) fluorescence spectrum given by a lognorm function[1]

$$f(\tilde{\nu}_{\mathrm{F}}) = \exp\left[-\ln(2)\left(\frac{\ln(1+2\gamma(\tilde{\nu}_{\mathrm{F}}-\tilde{\nu}_0)/\Delta)}{\gamma}\right)^2\right] \tag{1}$$

with the peak, width and asymmetry parameters $\tilde{\nu}_0$, Δ, and γ is converted into the expected upconverted spectrum $f(\tilde{\nu}_{\mathrm{U}})$ by multiplication with the spectral conversion efficiency $\eta(\tilde{\nu}_{\mathrm{F}})$

$$f(\tilde{\nu}_{\mathrm{U}}) = f(\tilde{\nu}_{\mathrm{F}})\,\eta(\tilde{\nu}_{\mathrm{F}}). \tag{2}$$

The spectral response of our setup is compared with two reference spectra. Data recorded at large delay times can be compared with the steady state spectrum, since the time-dependent Stoke's shift of the luminescence spectrum appears within a few picoseconds[4]. For this purpose, the steady state luminescence spectrum is fitted with a sum of two lognorm functions. According to equation (2), the fit is multiplied by the spectral efficiency curve of our setup to obtain the distribution of UV-photons expected in an upconversion measurement. The result is shown in Fig. S 3 as a grey dashed line together with the data measured 500 ps after excitation. Obviously the peak positions and the high energy parts of the spectra are in perfect agreement, while a small deviation is observed on the low energy side. In a second step, the luminescence spectrum shortly after excitation can be compared with values given in literature, which are obtained with a broadband upconversion scheme using a transmission geometry[1]. The parameters of a test fluorescence spectrum given by a lognorm function are varied until a best fit to the data measured 1 ps after excitation is achieved (blue dots in Fig. S 3). This procedure is done using spectral efficiency curves with (red dashed line in Fig. S 3) and without finite gate bandwidth. The fitting parameters are shown in Tab. S 1.

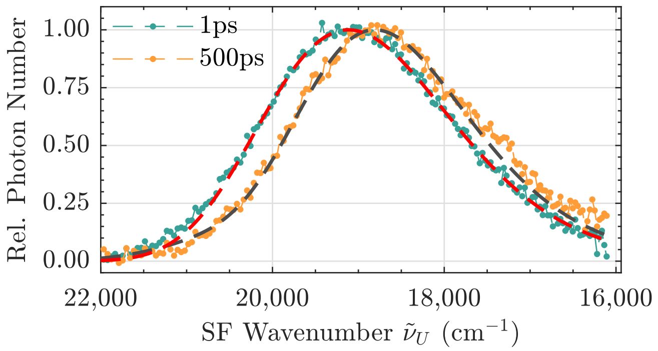

Figure S 3. Instantaneous luminescence spectra of Coumarin 153 solved in dimethyl-sulfoxide 1 ps (blue dots) and 500 ps (yellow dots) after excitation. The red line is a fit of an in silico upconverted lognormal function of equation (1) to the data, whereas the grey line reflects the steady state luminescence spectrum upconverted in silico.

Table S 1. Parameters obtained by fitting a lognorm function equation (1) to the luminescence spectrum of Coumarin 153 solved in dimethyl-sulfoxide 1ps after excitation. The photometric correction curve is calculated with and without consideration of the gate pulse bandwidth. The basic calculation using only the gate pulse central wavelength (Ref.[1]) is denoted with "monochr.". The extended calculation including the gate pulse bandwidth of ≈ 50 nm is denoted with "pulse". The first two rows compare parameters obtained with these two different calculation approaches. The third and fourth row give reference values reported in the literature[1], where the correction curves are calculated with a single gate wavelength.

	ν_0 (cm^{-1})	Δ (cm^{-1})	γ
pulse	19,000±150	3,300±200	-(0.3∓0.1)
monochr.	19,000±150	3,670±200	-(0.3∓0.1)
monochr.[1]	19,270	3,520	-0.29
	19,380	3,680	-0.34

It is evident that the parameters obtained by neglecting a finite gate bandwidth are very close to those given in literature, with the peak wavenumber being slightly smaller. This difference could stem from a lower coumarin concentration in Ref.[1], which leads to a lower self-absorption of the fluorescence in the blue spectral range. The table S 1 also shows that without taking the gate pulse width into account, the width of the spectrum is overestimated by $\approx 10\%$.

The striking agreement of our data with the steady state spectrum and results in the literature is remarkable. An additional experimental photometric correction using differently luminescing dyes as described in Ref.[1] is not required. This again proves that the theoretical photometric correction is very rigid, provided that the fluorescence and gate angles as well as the crystal thickness and gate pulse central wavelength are determined very thoroughly. For example, the angles are determined very accurately using the gate pulse reflected from the BBO crystal and a motorized rotation table. Pulses reflected directly back mark the gate angle zero. Then the crystal is rotated until the reflected gate pulse passes the off-axis Cassegrain reflector in such way that it is focused onto the sample, superimposing the pump pulse spot. The rotation angle now marks half the angle α. The gate pulse central wavelength and spectral width is characterized by a spectrometer sensitive to the near infrared spectral range (Ocean Optics *NIRQuest 512*). We like to note that the error margin given in Tab. S 1 reflects both the fitting error and the error in determining the angles used.

Additional optical elements in the beam path, such as the white glass filter used in the lithium niobate measurements, are also taken into account in the photometric correction by matrix multiplication.

Broadband Fluorescence Upconversion Spectroscopy on different lithium niobate samples

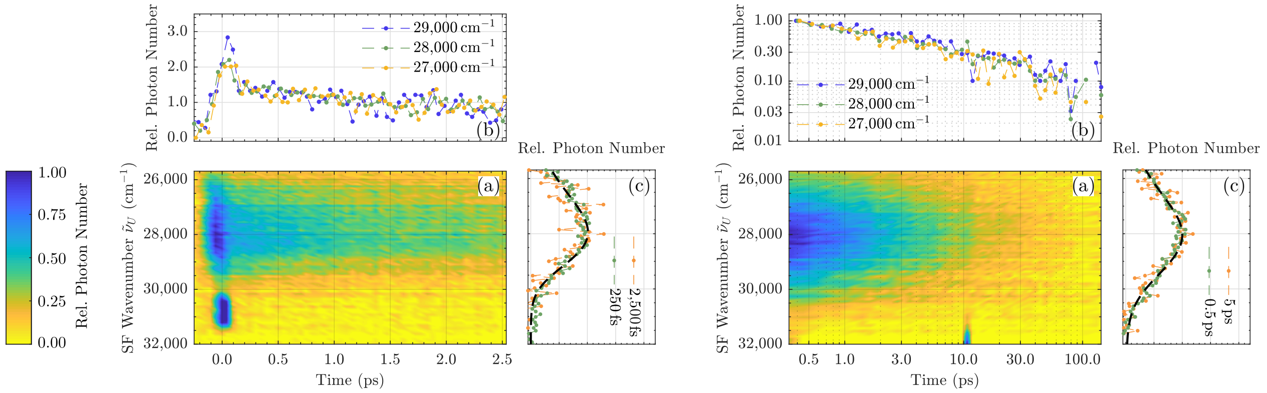

Figure S 4. (a) Upconverted luminescence spectra of undoped near-stoichiometric LN as a function of time. (b) Normalized kinetic trace for three selected wavenumbers. (c) Detailed view of the spectra normalized to their maximum value for two fixed delay times. The black dashed line is the steady state spectrum upconverted in silico (cf. dark yellow curve in Fig. 5 (b) in the manuscript). **Left:** Up to 2.5 ps, **Right**: Up to 200 ps.

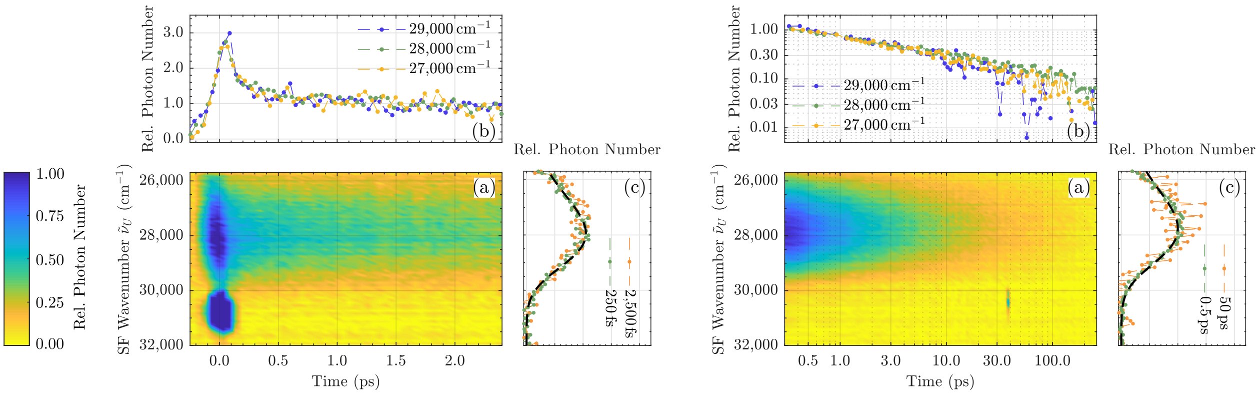

Figure S 5. (a) Upconverted luminescence spectra of Mg-doped (1 mol%) near-stoichiometric LN as a function of time. (b) Normalized kinetic trace for three selected wavenumbers. (c) Detailed view of the spectra normalized to their maximum value for two fixed delay times. The black dashed line is the steady state spectrum upconverted in silico (cf. dark blue curve in Fig. 5 (b) in the manuscript). **Left:** Up to 2.5 ps, **Right**: Up to 250 ps.

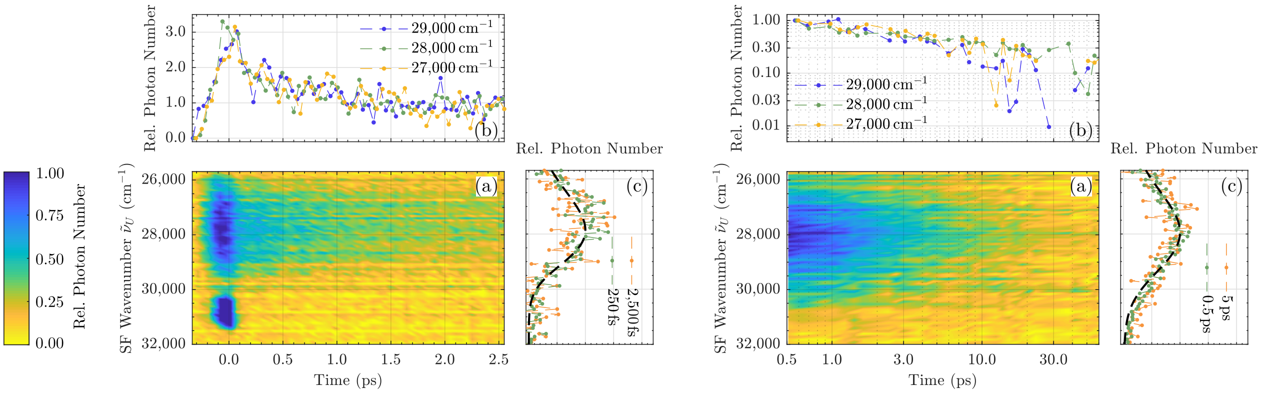

Figure S 6. (a) Upconverted luminescence spectra of Mg-doped (4.2 mol%) congruent LN as a function of time. (b) Normalized kinetic trace for three selected wavenumbers. (c) Detailed view of the spectra normalized to their maximum value for two fixed delay times. The black dashed line is the steady state spectrum upconverted in silico (cf. green curve in Fig. 5 (a) in the manuscript). **Left:** Up to 2.5 ps, **Right**: Up to 60 ps.

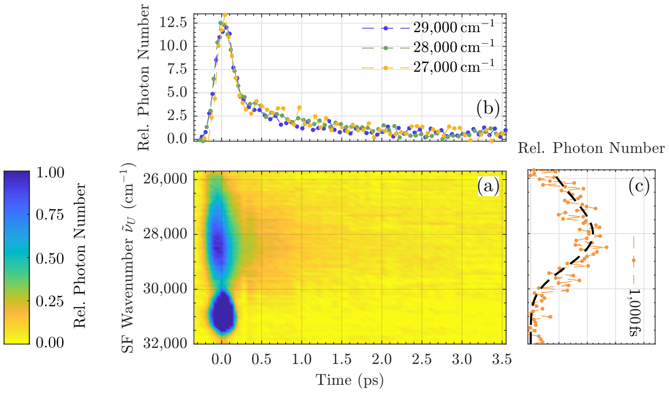

Figure S 7. (a) Upconverted luminescence spectra of undoped congruent LN as a function of time. (b) Normalized kinetic trace for three different wavenumbers. (c) Detailed view of the spectra normalized to their maximum value for two fixed delay times. The black dashed line is the steady state spectrum upconverted in silico (cf. yellow curve in Fig. 5 (a) in the manuscript)

References

1. Gerecke, M., Bierhance, G., Gutmann, M., Ernsting, N. P. & Rosspeintner, A. Femtosecond broadband fluorescence upconversion spectroscopy: Spectral coverage versus efficiency. *Rev. Sci. Instrum.* **87**, 053115 (2016).

2. Zhao, L., Lustres, J. L. P., Farztdinov, V. & Ernsting, N. P. Femtosecond fluorescence spectroscopy by upconversion with tilted gate pulses. *Phys. Chem. Chem. Phys.* **7**, 1716–1725 (2005).

3. Gerecke, M. *et al.* Effect of a Tertiary Butyl Group on Polar Solvation Dynamics in Aqueous Solution: Femtosecond Fluorescence Spectroscopy. *J. Phys. Chem. B* **121**, 9631–9638 (2017).

4. Martins, L. R., Tamashiro, A., Laria, D. & Skaf, M. S. Solvation dynamics of coumarin 153 in dimethylsulfoxide-water mixtures: Molecular dynamics simulations. *J. Chem. Phys.* **118**, 5955 (2003).
